# Supplementary material for: Analysis of chi angle distributions in free amino acids via multiplet fitting of proton scalar couplings
Source: Magn Reson (Gott). 2024 Aug 19;5(2):103–20. doi: 10.5194/mr-5-103-2024 (PMC11570886; doi:10.5194/mr-5-103-2024)
Supplement: The supplement related to this article is available online at: https://doi.org/10.5194/mr-5-103-2024-supplement. [file mr-5-103-2024-supplement.zip › Syed2024/data/fit1d_fitnmr_output.tar.gz!/fit1d/asp/400/bmse000031_simulation_1_nmredata/1H/resonances_1d.pdf]

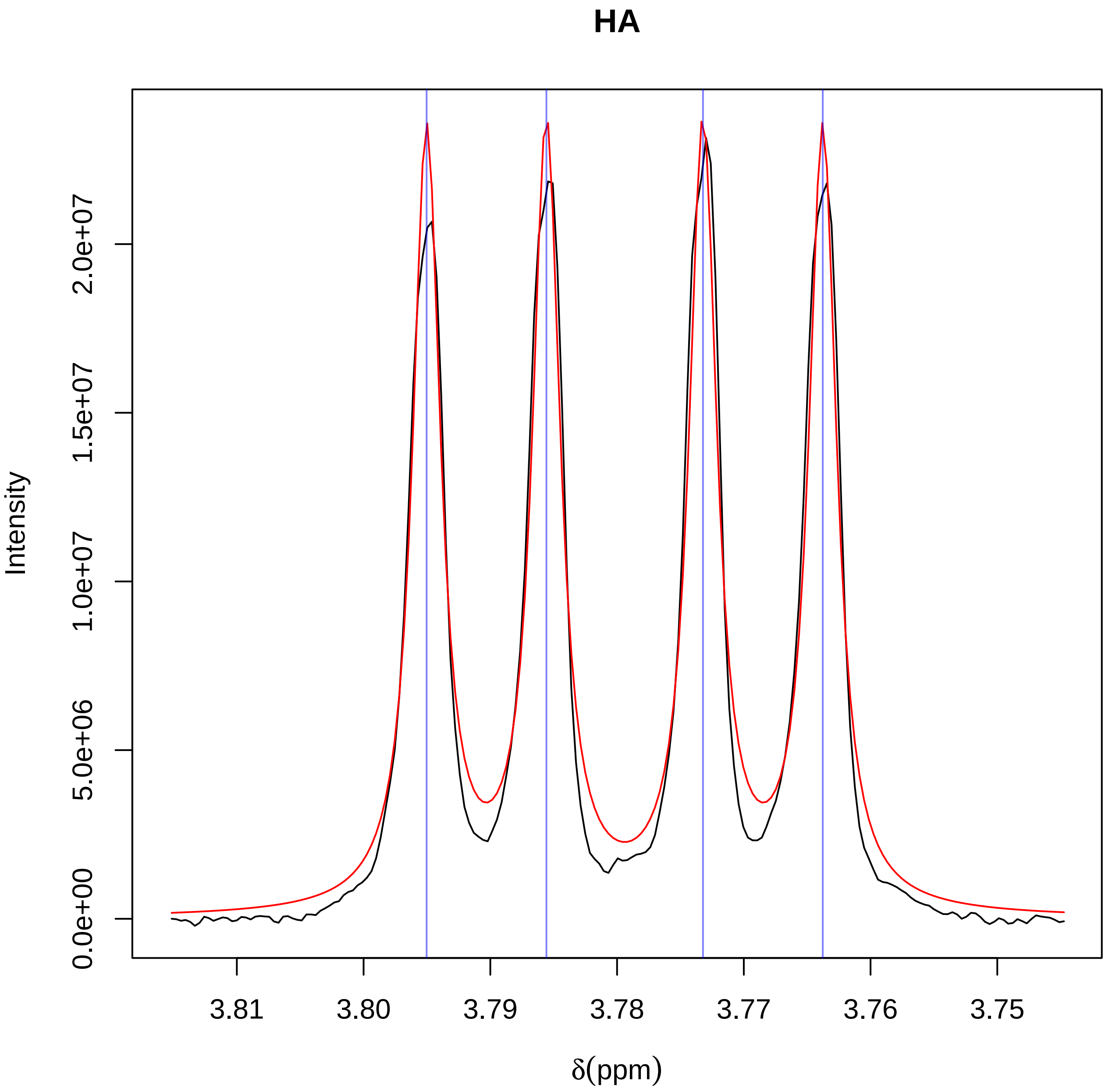

HA
Intensity
0.0e+00
5.0e+06
1.0e+07
1.5e+07
2.0e+07
3.81
3.80
3.79
3.78
3.77
3.76
3.75
δ(ppm)

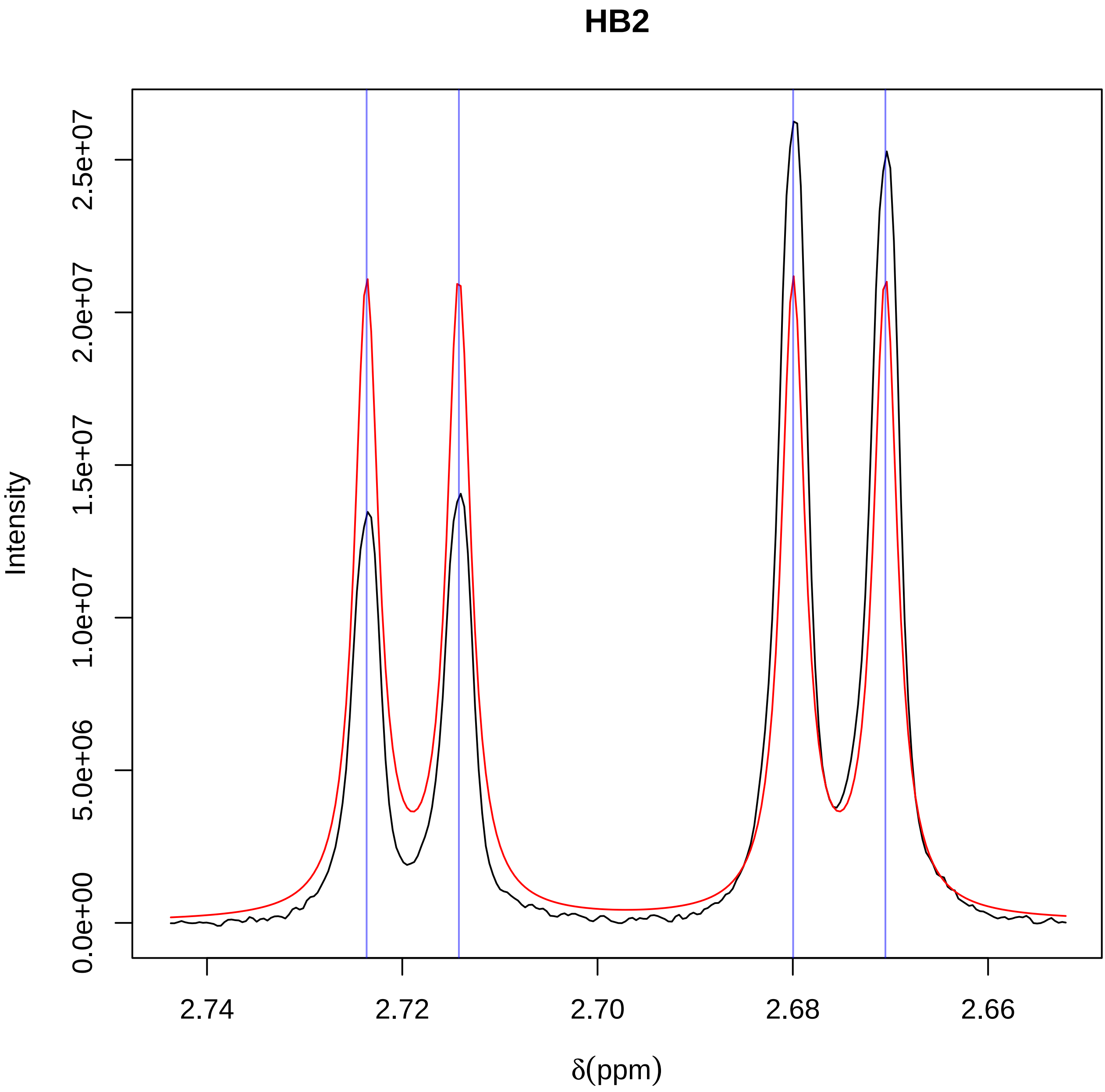
HB2
Intensity
0.0e+00
5.0e+06
1.0e+07
1.5e+07
2.0e+07
2.5e+07
2.74
2.72
2.70
2.68
2.66
δ(ppm)

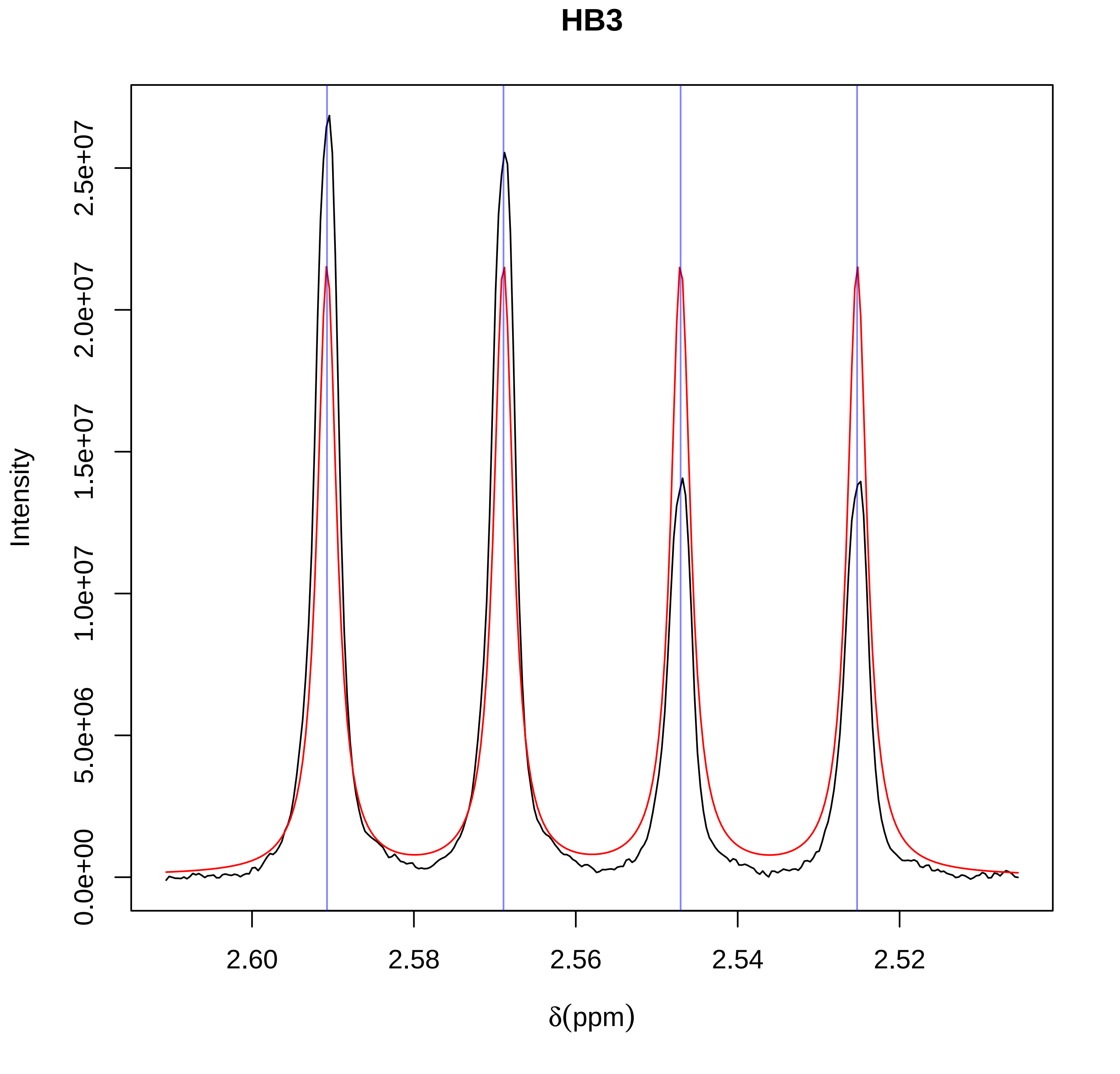

HB3
Intensity
0.0e+00
5.0e+06
1.0e+07
1.5e+07
2.0e+07
2.5e+07
2.60
2.58
2.56
2.54
2.52
δ(ppm)
